# The genomic landscape of dysembryoplastic neuroepithelial tumours and a comprehensive analysis of recurrent cases

**DOI:** 10.1111/nan.12834

**Published:** 2022-08-09

**Authors:** Mélanie Pagès, Marie‐Anne Debily, Frédéric Fina, David T. W. Jones, Raphael Saffroy, David Castel, Thomas Blauwblomme, Alice Métais, Marie Bourgeois, Emmanuèle Lechapt‐Zalcman, Arnault Tauziède‐Espariat, Felipe Andreiuolo, Fabrice Chrétien, Jacques Grill, Nathalie Boddaert, Dominique Figarella‐Branger, Rameen Beroukhim, Pascale Varlet

**Affiliations:** ^1^ GHU‐Paris – Sainte‐Anne Hospital, Department of Neuropathology Paris University Paris France; ^2^ Department of Genetics Institut Curie Paris France; ^3^ SIREDO Paediatric Cancer Center Institut Curie Paris France; ^4^ INSERM U830, Laboratory of Translational Research in Paediatric Oncology Institut Curie Paris France; ^5^ Paris Sciences Lettres Research University Paris France; ^6^ Molecular Predictors and New Targets in Oncology, INSERM U981, Gustave Roussy Université Paris‐Saclay Villejuif France; ^7^ Département de Biologie, Univ. Evry Université Paris‐Saclay Evry France; ^8^ APHM, CHU Timone Service d'Anatomie Pathologique et de Neuropathologie Marseille France; ^9^ Pediatric Glioma Research Hopp Children's Cancer Center (KiTZ) Heidelberg Germany; ^10^ Pediatric Glioma Research Group German Cancer Research Center (DKFZ) Heidelberg Germany; ^11^ Oncogenetics Department, Assistance Publique‐Hôpitaux de Paris, Paul Brousse Hospital Université Paris‐Saclay Villejuif France; ^12^ Pediatric Neurosurgery Department, AP‐HP Hôpital Universitaire Necker‐Enfants Malades Paris France; ^13^ Université de Paris‐ Cité Paris France; ^14^ Department of Neuropathology Instituto Estadual do Cérebro Paulo Niemeyer Rio de Janeiro Brazil; ^15^ Pathology Division, D'Or Research Institute (IDOR) D'Or Hospitals Network Rio de Janeiro Brazil; ^16^ Department of Pediatric and Adolescent Oncology Institut Gustave Roussy Villejuif France; ^17^ Pediatric Radiology Department, AP‐HP Hôpital Universitaire Necker‐Enfants Malades Paris France; ^18^ INSERM ERL UA10 Université de Paris Paris France; ^19^ Institut Imagine Université de Paris, UMR 1163 Paris France; ^20^ Institute of NeuroPhysiopatholy Aix‐Marseille Univ, CNRS, INP Marseille France; ^21^ Department of Medical Oncology Dana‐Farber Cancer Institute Boston Massachusetts USA; ^22^ Cancer Program Broad Institute Cambridge Massachusetts USA; ^23^ Department of Medicine Harvard Medical School Boston Massachusetts USA

**Keywords:** DNA methylation profiling, dysembryoplastic neuroepithelial tumours, *FGFR1*, glioneuronal tumours, molecular pathology, paediatric low‐grade gliomas

## Abstract

**Aims:**

Dysembryoplastic neuroepithelial tumour (DNT) is a glioneuronal tumour that is challenging to diagnose, with a wide spectrum of histological features. Three histopathological patterns have been described: specific DNTs (both the simple form and the complex form) comprising the specific glioneuronal element, and also the non‐specific/diffuse form which lacks it, and has unclear phenotype–genotype correlations with numerous differential diagnoses.

**Methods:**

We used targeted methods (immunohistochemistry, fluorescence in situ hybridisation and targeted sequencing) and large‐scale genomic methodologies including DNA methylation profiling to perform an integrative analysis to better characterise a large retrospective cohort of 82 DNTs, enriched for tumours that showed progression on imaging.

**Results:**

We confirmed that specific DNTs are characterised by a single driver event with a high frequency of *FGFR1* variants. However, a subset of DNA methylation‐confirmed DNTs harbour alternative genomic alterations to *FGFR1* duplication/mutation. We also demonstrated that a subset of DNTs sharing the same *FGFR1* alterations can show in situ progression. In contrast to the specific forms, “non‐specific/diffuse DNTs” corresponded to a heterogeneous molecular group encompassing diverse, newly‐described, molecularly distinct entities.

**Conclusions:**

Specific DNT is a homogeneous group of tumours sharing characteristics of paediatric low‐grade gliomas: a quiet genome with a recurrent genomic alteration in the RAS‐MAPK signalling pathway, a distinct DNA methylation profile and a good prognosis but showing progression in some cases. The “non‐specific/diffuse DNTs” subgroup encompasses various recently described histomolecular entities, such as PLNTY and diffuse astrocytoma, *MYB* or *MYBL1* altered.

## Graphical abstract

Specific DNTs are characterized by a single driver event with a high frequency of *FGFR1* duplication/mutation, but alternative genomic alterations could be observed. A subset of DNTs sharing the same *FGFR1* alterations can show *in situ* progression. DNA methylation profiling could convey prognostic information as DNT‐MC‐1 is enriched with progressive form of DNT. Contrarily to specific forms, “non‐specific/diffuse DNTs” corresponded to a heterogeneous molecular group encompassing newly described distinct histo‐molecular tumours as PLNTY or diffuse astrocytoma, *MYB* or *MYBL1*‐altered.

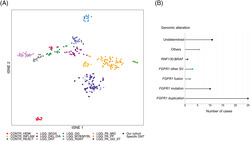

**List of abbreviations**
CD34: haematopoietic progenitor cell antigen CD34
cIMPACT‐NOW: consortium to inform molecular and practical approaches to CNS tumour taxonomy**‐**not official WHO
CNS: central nervous system
CNV: copy number variation
ddPCR: droplet digital polymerase chain reaction
DLGNT: diffuse leptomeningeal glioneuronal tumour
DNT: dysembryoplastic neuroepithelial tumour
FFPE: formalin‐fixed‐paraffin embedded
FISH: fluorescence in situ hybridisation
HES: haematoxylin‐eosin‐saffron
IHC: immunohistochemistry
LGG: low‐grade glioma
LGGNT: low‐grade glioneuronal tumour
MAPK: mitogen‐activated protein kinase
MRI: magnetic resonance imaging
NOS: not otherwise specified
PLNTY: polymorphous low‐grade neuroepithelial tumour of the young
TKD: tyrosine kinase domain
t‐SNE: t‐distributed stochastic neighbour embedding
WES: whole‐exome sequencing
WHO: world health organisation

**Key Points**

- DNTs are characterised by *FGFR1* alterations, including single nucleotide variations and gene rearrangements, but the lack of *FGFR1* disruption is not sufficient to rebut a diagnosis of DNT.
- As occasionally observed in glioneuronal tumours, pilocytic astrocytomas or paediatric‐type diffuse low‐grade gliomas, DNTs can progress (over many years) through iterative surgeries and require a long‐term follow‐up. Such progression does not signify malignant transformation and should not necessarily question the diagnosis of DNT.
- DNA methylation profiling can help in diagnosing challenging DNT cases and may convey prognostic information at diagnosis on the progression risk.
- The terms “non‐specific” and “diffuse DNT” mentioned in the WHO CNS tumours classification correspond to a heterogeneous molecular group encompassing diverse and newly described distinct glioneuronal tumours and paediatric‐type diffuse low‐grade gliomas.

## INTRODUCTION

Paediatric diffuse low‐grade gliomas (LGG) and low‐grade glioneuronal tumours (LGGNT) account for 25–30% of all central nervous system (CNS) tumours of childhood [1, 2]. This large group of tumours is highly heterogeneous, causing major diagnostic challenges, and also encompasses newly recognised types in the fifth edition of the World Health Organisation (WHO) CNS tumours, such as diffuse astrocytoma, MYB/MYBL1 altered, polymorphous low‐grade neuro‐epithelial tumour of the young and diffuse low‐grade, MAPK pathway‐altered. LGGNTs, including dysembryoplastic neuroepithelial tumours (DNTs), are particularly challenging to diagnose since this group includes a large spectrum of tumours that are difficult to discriminate based on their histopathological features. DNTs are characterised by a cortical location and their association with early‐onset drug‐resistant focal epilepsy and account for 5 to 20% of histopathological diagnoses in epilepsy surgery depending on the histopathological criteria used [3, 4, 5]. The histopathological hallmarks are a multinodular growth pattern and the specific glioneuronal element, which is observed in specific DNTs. The simple form consists of the unique glioneuronal element, and the complex one consists of the glioneuronal element in combination with glial nodules. Non‐specific DNTs lack the specific glioneuronal element, rendering them difficult to diagnose, and although this entity was described in the 2016 WHO classification, it remains under debate [6].

The genomic profile of DNTs has been shown to be stable, with only a few copy number alterations. Mutation or duplication of the tyrosine kinase domain of *FGFR1* have been found in approximately 70% of DNTs, whilst recurrent *BRAF* V600E mutations have been identified in approximately 30% of the DNTs, including all subtypes [7, 8, 9, 10]. However, most of these studies were performed before the era of DNA methylation profiling.

DNTs are WHO grade I tumours and classically depicted as stable, even in the event of partial surgical resection. Their prognosis depends primarily on epilepsy‐related morbidity [11, 12, 13, 14]. Nevertheless, some cases of progressive tumours or those showing malignant transformation have been described in the literature [15, 16, 17, 18, 19, 20, 21, 22, 23, 24, 25, 26]. However, the immunohistochemical features of these cases were succinct, and molecular data were extremely scarce or non‐existent, so that the diagnosis of DNT could legitimately be challenged. Indeed, very limited molecular information is available on progressive DNTs, and so far, there is no series that included molecular data characterising these rare circumstances.

We used targeted methods (immunohistochemistry (IHC), fluorescence in situ hybridisation (FISH) and targeted sequencing), and large‐scale genomic and epigenetic methodologies to perform integrative analyses with histology and imaging data to further characterise specific and “non‐specific” DNTs, including specific DNTs that went on to progress (“progressive DNTs”).

## MATERIALS AND METHODS

### Study design

All samples were first subjected to histopathological review (MP, PV). Complex DNTs and non‐specific DNTs were screened for IDH1 R132H and H3K27M substitutions by IHC. All samples were then subjected to DNA methylation profiling and targeted analyses for the most common alterations described in DNT: *FGFR1* mutations in exons 12 and 14, *FGFR1* internal‐tandem duplication (ITD) and BRAF V600E mutations, using IHC and droplet digital polymerase chain reaction (ddPCR, supporting information Figure S1). Since gene rearrangements are a frequent event in paediatric LGGNT, negative cases with frozen tissue available were submitted for RNA sequencing analyses to screen for gene fusions. When no frozen tissue was available, samples were subjected to FISH analysis using break‐apart probes targeting the four most frequent gene rearrangements in paediatric LGNT (*BRAF, FGFR2, MYB* and *MYBL1*). Finally, samples without an identified potential driver alteration were submitted for more extensive sequencing: whole‐exome sequencing (WES) for samples with frozen tissue and targeted sequencing of a panel of 22 cancer‐relevant genes for samples with only formalin‐fixed‐paraffin embedded (FFPE) material (supporting information Figure S1).

### Patients and tumour samples

We screened the GHU‐Paris‐Sainte‐Anne hospital neuropathology longitudinal database for patients diagnosed with DNT from January 1993 to December 2016 allowing us to retrieve 217 tumours. After patient consent collection as well as assessment for suitable tissue availability (FFPE or frozen tissue), 172 tumours were eligible. Subsequently excluding the tumours with no available clinical or magnetic resonance imaging (MRI) data and those for which the retrieved material did not allow further histomolecular investigation (poor tissue quality and failed DNA/RNA extraction), we obtained a cohort of 112 tumours. We selected 82 tumours that met criteria for the diagnosis of specific and non‐specific DNT according to the current WHO CNS tumour classification. We limited the inclusion of “classic” DNT, which have been previously well characterised in the literature (30 “classic” DNT were excluded from this analysis), with the aim to focus our efforts on characterising recurrent DNTs. Therefore, the cohort was enriched with DNTs that went on to progress after surgery (called “progressive DNTs”) and was not composed of consecutive cases. Clinical data were collected from the patient records, including sex, age at diagnosis, tumour location and seizure history. Imaging review was performed under the supervision of a senior paediatric neuroradiologist (NB). Pre‐ and post‐operative MRIs were compared to evaluate the presence of a post‐operative residue, and to evaluate tumour‐size increase and contrast enhancement occurrence. Progressive DNT was defined as a DNT showing at least one of the following three criteria during the follow‐up: an increased size of hypoT1/hyperT2 signal and/or an increased size of the contrast enhancement and/or the occurrence of contrast enhancement.

Sections for genomic analyses and IHC were prepared from zinc formalin‐fixed paraffin‐embedded tissue specimens (5% formalin, 3 g/L zinc, 8 g/L sodium chloride) or frozen tissues with a paired‐smear control for tumour cell content estimation. All patients consented to participation in the Necker hospital tumour bank, and the study received ethical committee approval (ID‐RCB 2017‐A01535‐48). The Kaplan–Meier analyses were performed for survival data using the log‐rank test. The level of significance was *p* < 0.05. Analyses were performed using Prism software (v9.1.2).

### Immunohistochemistry

Representative zinc formalin‐fixed sections were deparaffinised and processed with a Ventana autostainer (BenchMark XT, Ventana Medical Systems or Discovery XT, Ventana Medical Systems) according to a standard protocol. Antibodies against the following proteins were used: CD34 (1:40, QBEnd‐10, Dako, Denmark A/S, Glostrup, Denmark), chromogranin A (1:200, LK2H10, Diagnostic BioSystems, Pleasanton, USA), p53 (1:5000, DO‐1, Santa Cruz Biotechnology, Dallas, USA), ATRX (1:200, polyclonal, Sigma Aldrich, St. Louis, MO), BRAF V600E (1:100, VE1, Spring Bioscience, Pleasanton, USA), H3K27M (1:1000, ABE419, EMD Millipore, Billerica, USA), H3K27me3 (1:1250, C15410195, Diagenode, Seraing, Belgium), IDH1 R132H (1:35, H09, Dianova, Hamburg, Germany) and CIC (1:250, 6E12.1, EMD Millipore, Billerica, USA). Antibody binding was detected by incubation with the chromogen diaminobenzidine. The slides were then scanned in a NanoZoomer 2.0‐RS (Hamamatsu Photonics, Hamamatsu, Japan).

### Droplet digital PCR

An area representative of tumour was selected from haematoxylin‐eosin‐saffron (HES) stained sections, and the tumour cell content was estimated for each sample. Tumour DNA was extracted from 4‐μm thick sections of zinc formalin‐fixed paraffin‐embedded tissue.

*BRAF* V600E mutation, *BRAF* exon 14 duplication, *FGFR1* N546K/K656E mutations and *FGFR1* TKD duplication were assessed for every patient by previously described droplet digital PCR (ddPCR) [27, 28, 29]. Fractional abundance and copy number variation (CNV) were calculated with the cut‐off values and detection thresholds defined by Appay et al. [28].

### Fluorescence in situ hybridisation

FISH analysis was performed on interphase nuclei on paraffin‐embedded tissue (4 μm), following standard procedures as previously described [30, 31] and using break‐apart probes targeting *BRAF*, *FGFR2*, *MYB*, *MYBL1* and *FGFR3*. A case was considered positive when the scored nuclei displayed a break‐apart signal in at least 20% of the counted nuclei. Hybridisations were considered non‐informative if the FISH signals were either lacking or too weak to be interpreted. The results were recorded using a DM6000 imaging fluorescence microscope (Leica Biosystems, Nanterre, France) fitted with appropriate filters, a CCD camera and digital imaging software (CytoVision, v7.4).

### Targeted sequencing

An area representative of the tumour was selected from HES sections, and the tumour cell content was estimated for each sample. Tumour DNA was extracted from 4‐μm thick sections of FFPE tissue using QIASymphony kit (QIAGEN, Hilden, Germany) according to the manufacturer's recommendations. Libraries were constructed using xGen Lockdown IDT probe‐Sophia Genetics (SOPHiA GENETICS, Saint‐Sulpice, Switzerland) and sequenced on a MiSeq (Illumina, San Diego, CA) to minimal genome‐wide fold coverage of 500X. Sequence reads were mapped to the human genome build (hg19), and analyses were performed using SophiaDDMTM bioinformatic platform (SOPHiA GENETICS, Saint‐Sulpice, Switzerland). Genes targeted were *AKT, ALK, BRAF, CDK4, CDKN2A, CTNNB1, DDR2, DICER1, EGFR, ERBB2, FBXW7, FGFR1, FGFR2, FGFR3, KRAS, MAP 2 K1, MET, MYOD1, NRAS, PDGFRA, PIK3CA, PTPN11, RAC1, RAF1, RET, ROS1, FOXL2, GNA11, GNAQ, GNAS, H3F3A, H3F3B, HIST1H3B, HRAS, IDH1/2, KIT, SF3B1, SMAD4, TERT* and *TP53*.

### RNA sequencing

Total RNA was extracted from frozen tissue using the Allprep® DNA/RNA extraction mini kit from Qiagen (Hilden, Germany) according to the manufacturer's recommendations and quantified on a Bioanalyzer (Agilent Technologies). Library preparation and RNA sequencing were performed by IntegraGen SA (Evry, France). Libraries were prepared using the TruSeq Stranded mRNA sample preparation kit (Illumina) following the supplier's recommendations. Following mRNA purification, the RNA was chemically fragmented prior to reverse transcription and cDNA generation. The cDNA fragments then went through an end repair process, the addition of a single “A” base to the 3′ end and then ligation of the adapters. Finally, the products were purified and enriched with PCR to create the final double stranded cDNA library, which was then purified and quantified by quantitative polymerase chain reaction (QPCR). Sequencing was performed using 75bp paired‐end runs on Illumina HiSeq 4000.

Candidate fusion genes were identified using FusionCatcher (V.0.99.7c) and Star‐Fusion (V.0.8.0). Then, in silico validation of a list of fusion transcripts prediction was done by FusionInspector, a component of the Trinity Cancer Transcriptome Analysis Toolkit.

### Whole exome sequencing

Total tumour DNA was extracted from frozen tissue and matched normal DNA was extracted from blood using the Allprep® DNA/RNA extraction mini kit from Qiagen (Hilden, Germany) according to the manufacturer's recommendations. Library preparation, exome capture, sequencing and data analysis were performed by IntegraGen SA (Evry, France). Sequence capture, enrichment and elution were performed using Agilent in‐solution enrichment methodology (SureSelect clinical research exome v2, Agilent) according to the manufacturer's instruction and protocols without modification except for library preparation performed with NEBNext® Ultra kit (New England Biolabs®). For library preparation, 150 ng of each genomic DNA was fragmented by sonication and purified to yield fragments of 150–200 bp. Paired‐end adaptor oligonucleotides from the NEB kit were ligated on repaired A tailed fragments and then purified and enriched by 7 PCR cycles. Seventy‐hundred nanogram of these purified libraries were then hybridised to the SureSelect oligo probe capture library for 24 h. After hybridisation and washing, captured products were PCR amplified with 9 cycles, purified and quantified by QPCR to obtain sufficient DNA template for downstream applications. Sequencing was performed using 75 bp paired‐end runs on Illumina NextSeq 500. Mean depth expected was 60x for germinal DNA and 110x for tumour DNA.

Base calling was performed using the Real‐Time Analysis software sequence pipeline with default parameters. Sequence reads were mapped to the human genome build (hg38) using the Burrows‐Wheeler Aligner tool. The duplicated reads were removed (sambamba tools). Variant calling, allowing the identification of genetic alterations as well as single‐nucleotide variant (SNV), small insertions/deletions (up to 20 bp) was performed using Broad Institute's GATK Haplotype Caller Genomic Variant Call Format tool (3.7) for constitutional DNA and Broad Institute's MuTect tool for somatic DNA. In‐house postprocessing was applied to filter out candidate somatic mutations that were more consistent with artefacts or germline mutations. Ensembl's Variant Effect Predictor (release 90, GENCODE 27) programme processed variants to evaluate the effect on relevant transcripts and proteins and predict the functional consequences of variants (based on data available in dbSNP (dbSNP150), 1000 Genomes Project (1000G_phase3), Exome Variant Server (ESP6500SI‐V2‐SSA137), Exome Aggregation Consortium (ExAC r3.0) and our in‐house databases). Regarding missense changes, we used bioinformatics predictions for pathogenicity SIFT (v5.2.2), PolyPhen (v2.2.2), PANTHER (v13.1) and MutationAssessor. To investigate genomic copy number aberrations (CNA), we used the Bioconductor DNACopy package (DNAcopy 1.32.0) by comparing the normal DNA exome data to a reference samples pool. All changes were annotated with the Genomic Variants database. B‐allele frequency was investigated using the Bioconductor DNACopy package (DNAcopy 1.32.0) with mutated allele frequencies of SNVs known in 1000 Genomes Project.

### Methylation analysis and data processing

DNA‐methylation profiling was performed at the DKFZ Genomics and Proteomics Core Facility (Heidelberg, Germany) utilising the Illumina HumanMethylation450 BeadChip array (450 k array or EPIC) (Illumina, San Diego, USA) according to the manufacturer's instructions and as previously reported [32]. All samples were checked for expected and unexpected genotype matches.

The .idat files were uploaded to the online CNS tumour DNA methylation classifier at https://www.molecularneuropathology.org (v11b4) and a report for every tumour was generated, providing prediction scores for methylation classes and chromosomal copy‐number plots. The calibrated scores were integrated in the histopathological findings according to the recommendations from Capper et al. [33] and as previously reported [34].

Additional analyses were performed in R studio (v4.0.2). Raw signal intensities were obtained from .idat files using the minfi Bioconductor package (v1.34.0). Background correction and dye‐bias correction were performed on each sample. A correction for the type of specimen (FFPE or frozen) was performed with the removeBatchEffect function (limma package v3.44.3). Filtering of probes was performed using several criteria: removal of probes targeting X or Y chromosomes, removal of probes containing single nucleotide polymorphisms and probes not included in the EPIC array.

t‐SNE (t‐Distributed Stochastic Neighbour Embedding) was performed using the Rtsne package (v0.15). We selected the most variable probes for t‐SNE (SD > 0.20) with parameter theta = 0, pca = TRUE, max_iter = 2500 and perplexity = 10, based on the method reported by Capper et al. [32]. Two‐hundred and forty‐four CNS tumours corresponding to nine methylation classes from the Heidelberg reference cohort were included. Hierarchical clustering was performed using the Complex Heatmap package (2.4.3). Clustering of the most variable probes (SD > 0.15) from methylation arrays was performed based on Euclidean distance with the Ward's method.

Chromosomal CNV were analysed using plots generated by the MNP website https://www.neuropathology.org as well as by generating additional plots using the conumee R package (v1.22.0). Focal CNV observed on the plots were investigated using the segmented files and the Integrative Genomics Viewer visualisation. All CNV plots were also checked for noise.

All other plots and graphs were generated using the ggplot2 (v3.3.2) package in R studio (v4.0.2).

## RESULTS

### Clinical and histopathological data of the cohort

We retrieved 82 paediatric and young adult tumours diagnosed as DNT from the neuropathological archives of GHU‐Paris‐Sainte‐Anne hospital, including 51 males and 31 females, with a median age at diagnosis of 10 years (range 2–29) (Table 1 and supporting information Tables S1 and S2).

**TABLE 1:** Clinical and histopathological characteristics of the DNT cohort

	Specific DNT			
	*N* = 58			
	Simple form	Complex form	Nonspecific DNT	Total
	*n* = 13	*n* = 45	*N* = 24	*N* = 82
**Gender F/M** (*N* = 82)	0.6	0.45	1	0.6
**Median age at 1st surgery** (*N* = 82)	7	10	8.5	10
**Location** (*N* = 82)				
Frontal	4	10	7	21 (26%)
Temporal	4	18	14	36 (45%)
Parietal	2	7	0	9 (11%)
Occipital	2	2	1	5 (6%)
Fronto‐temporal	0	2	0	2 (2%)
Temporo‐parietal	0	2	0	2 (2%)
Temporo‐occipital	0	0	2	2 (2%)
Parieto‐occipital	1	2	0	3 (4%)
Temporo‐parieto‐occipital	0	1	0	1 (1%)
Septal	0	1	0	1 (1%)
**Extravascular CD34 +** (*N* = 82)	0	6 (13%)	10 (42%)	16 (20%)

After histopathological review, a specific glioneuronal component was observed in 58 cases (specific DNTs), and 13 and 45 cases were classified as simple and complex DNTs according to the WHO classification criteria. Other cases (*n* = 24) were classified as “non‐specific” DNT according to the WHO classification and further analysed separately from specific DNTs (Table 1 and supporting information Tables S1 and S2).

### Histomolecular characteristics of the specific DNTs cohort

By IHC, among the 58 cases of specific DNTs, no simple DNT showed extravascular CD34 expression and only 6 (13%) complex DNTs did (Table 1 and supporting information Table S1). None of the samples exhibited IDH R132H, H3 K27M alterations, or loss of H3K27 trimethylation resulting from K27M mutations.

**FIGURE 1:**
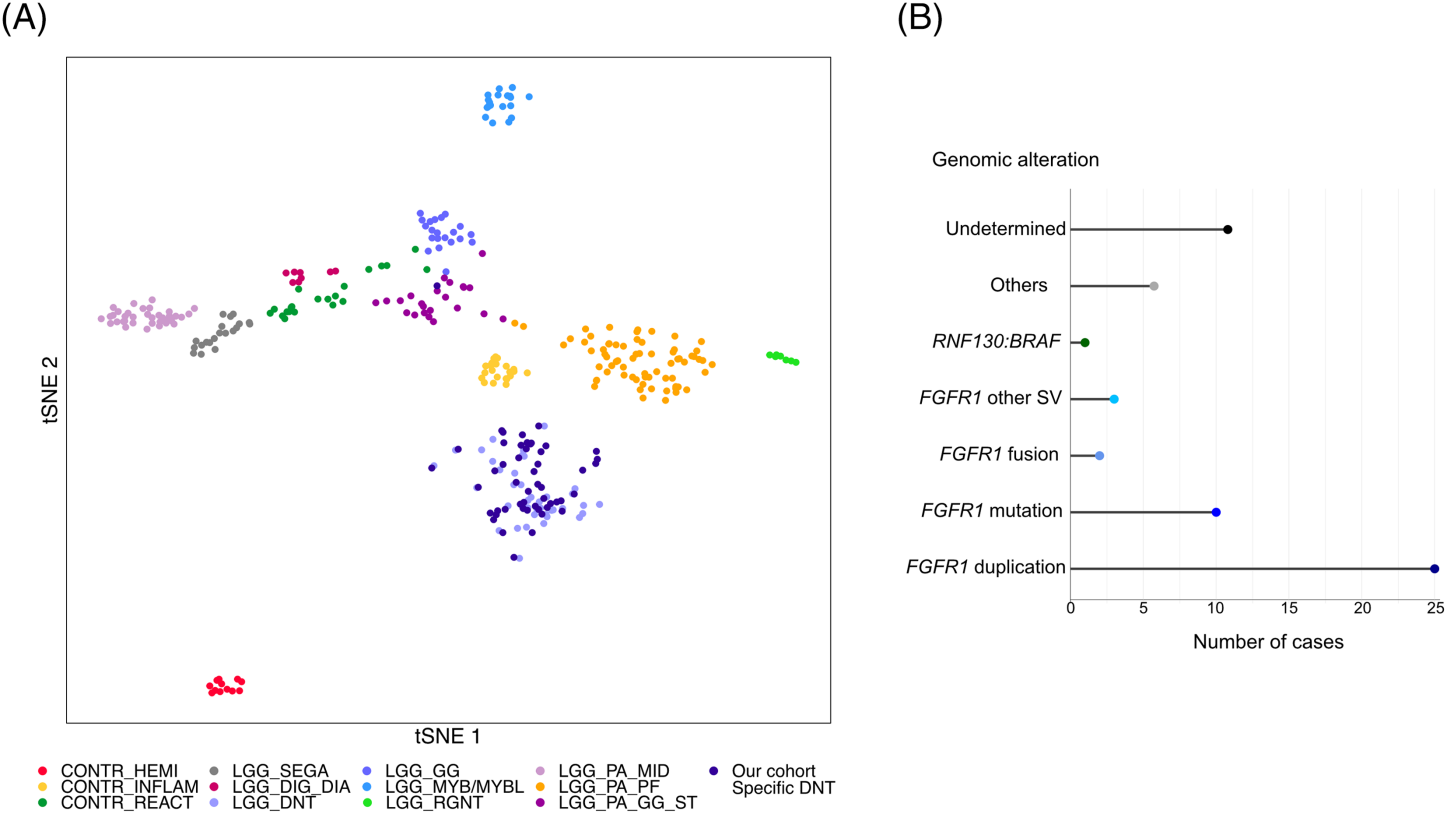
(A) **Classification of 51 DNTs in the paediatric tumour DNA methylation landscape**. The 51 DNTs with DNA methylation data available were compared with 244 reference low‐grade gliomas samples cohort belonging to 9 methylation classes and 74 control samples from the German Cancer Research Center (DKFZ) [32]. The 51 cases of this study are indicated as dark purple dots. (B) **Genomic alterations identified in the 58 DNTs of the cohort**

Of the 58 specific DNTs, DNA methylation profiling was available for 51 patients (62 samples/88%, seven failed). DNA methylation profiling was performed at diagnosis for 42/51 patients (82%). Of these 42 tumours, 37 (88%) were associated with a calibrated score >0.9 allowing a methylation class assignment with certainty; all were classified into the “LGG, DNT” methylation group. DNA‐methylation profiling failed to classify with high confidence five cases (three cases with a calibrated score ranging from 0.58 to 0.78 for LGG_DNT and two cases with a calibrated score <0.3). For nine additional patients (11 samples analysed), a methylation class was only obtained from samples obtained from the progression; among them, five fell into the “LGG, DNT” methylation group with a calibrated score >0.9, whereas DNA‐methylation profiling failed to classify with certainty four cases (two cases with a calibrated score of 0.68 and 0.79 for LGG_DNT and two cases with a calibrated score <0.3) (supporting information Tables S1 and S3). A two‐dimensional *t*‐stochastic neighbour embedding (t‐SNE) projection alongside 244 LGGs and LGGNTs from the Heidelberg reference cohort showed that all samples clustered with DNTs from the reference cohort and separately from other LGGs and LGGNTs, including those that were associated with a low calibrated score, with the exception of one case that clustered with the LGG PA/GG ST methylation class (Figure 1A).

Mutations or structural variations (SVs) involving *FGFR1* were found in 37/51 of the DNA methylation profiled tumours (73%), including 23 ITDs, 9 mutations, 2 fusions and 3 other SVs (Figure 1B and supporting information Table S1). All *FGFR1*‐mutated DNTs harboured the hotspot mutation at residue K656 with the exception of one harbouring a mutation at residue N546, another hotspot. In addition, two cases harboured a second point mutation in *FGFR1*, H649R and D652G. No *FGFR1* status was available for two patients for whom ddPCR failed, and no frozen tissue was available for further analyses (old samples, >10 years). Among the seven tumours without available methylation profiling, *FGFR1* status was obtained for six (two ITDs, one mutation and three wild type) (Figure 1B and supporting information Table S1).

All 58 specific DNTs of the cohort have been screened for BRAF V600E by IHC and/or ddPCR. No BRAF V600E mutation was observed.

We performed RNA sequencing in 14 cases and whole exome sequencing in 6 cases lacking *FGFR1* disruption and for which frozen tissue was available (supporting information Tables S1 and S4), as well as targeted DNA sequencing for those with no frozen tissue available. We detected one fusion, and mutations in various genes across this panel of tumours, but none of these alterations were recurrent. However, several genes are known to be frequently involved in genomic events in other cancers, including paediatric LGGs, and even have been shown to play driver roles. A fusion involving *BRAF* was detected in one tumour (DNA methylation score of 0.58 for LGG‐DNT) and represented the unique *BRAF* disruption identified in this cohort of specific DNTs (Figure 1B). The fusion partner was *RNF130*, previously reported in ganglioglioma, pilocytic astrocytoma and DNT [35, 36]. One case harboured a mutation in the proto‐oncogene *YES1*, encoding Src family tyrosine kinase. A missense mutation leading to K1465N substitution in *MTOR* was identified in one case. One case was characterised by the presence of a copy gain on chr22q (1 Mb). Interestingly, two cases harboured mutations in *IDH1/2* genes; *IDH1* A51T of undetermined significance has not been previously described whilst *IDH2* R140W is a hotspot mutation. One case stood out by the presence of two germline mutations, involving *BRCA2* (DNA repair) and *FGFR3* (tyrosine‐protein kinase receptor). The nonsense *BRCA2* R2318* mutation has been highly reported and is associated with very strong evidence of pathogenicity. We detected a *CIC* S1552R substitution in one case, previously reported in one oligodendroglioma III (COSS2375605); however, IHC did not show loss of expression of CIC. We indicate which platform was used for each tumour in supporting information Tables S1 and S4. Supporting information Table S4 and Figure S2 summarise the molecular findings.

### Integrative analysis of progressive specific DNT

We subsequently focussed our analysis on 25 specific DNTs, all with a DNA methylation profile and with post‐surgical progression documented on MRI (called “progressive DNTs”).

The median age at first surgery was 9 years old (range 2–16). The sex ratio F/M was 0.8. The median follow‐up was 7 years (range 1–27), and the median between the first and the second operation was 5 years (range 1–9). Interestingly, contrast enhancement MRI images were available for 22 tumours, with a high proportion of cases showing contrast enhancement during the follow‐up (19/22, 86%). This included 12/21 (57%) with contrast enhancement at diagnosis, whilst only 4/25 of the non‐progressive specific DNTs (16%) showed contrast enhancement at diagnosis.

Among the progressive specific DNTs, 17 tumours (68%) harboured an *FGFR1* disruption, including 10 ITDs, 5 mutations, 1 fusion and 1 other alternative SV. No recurrent genomic alteration was specifically identified in progressive specific DNTs (supporting information Tables S1 and S4).

**FIGURE 2:**
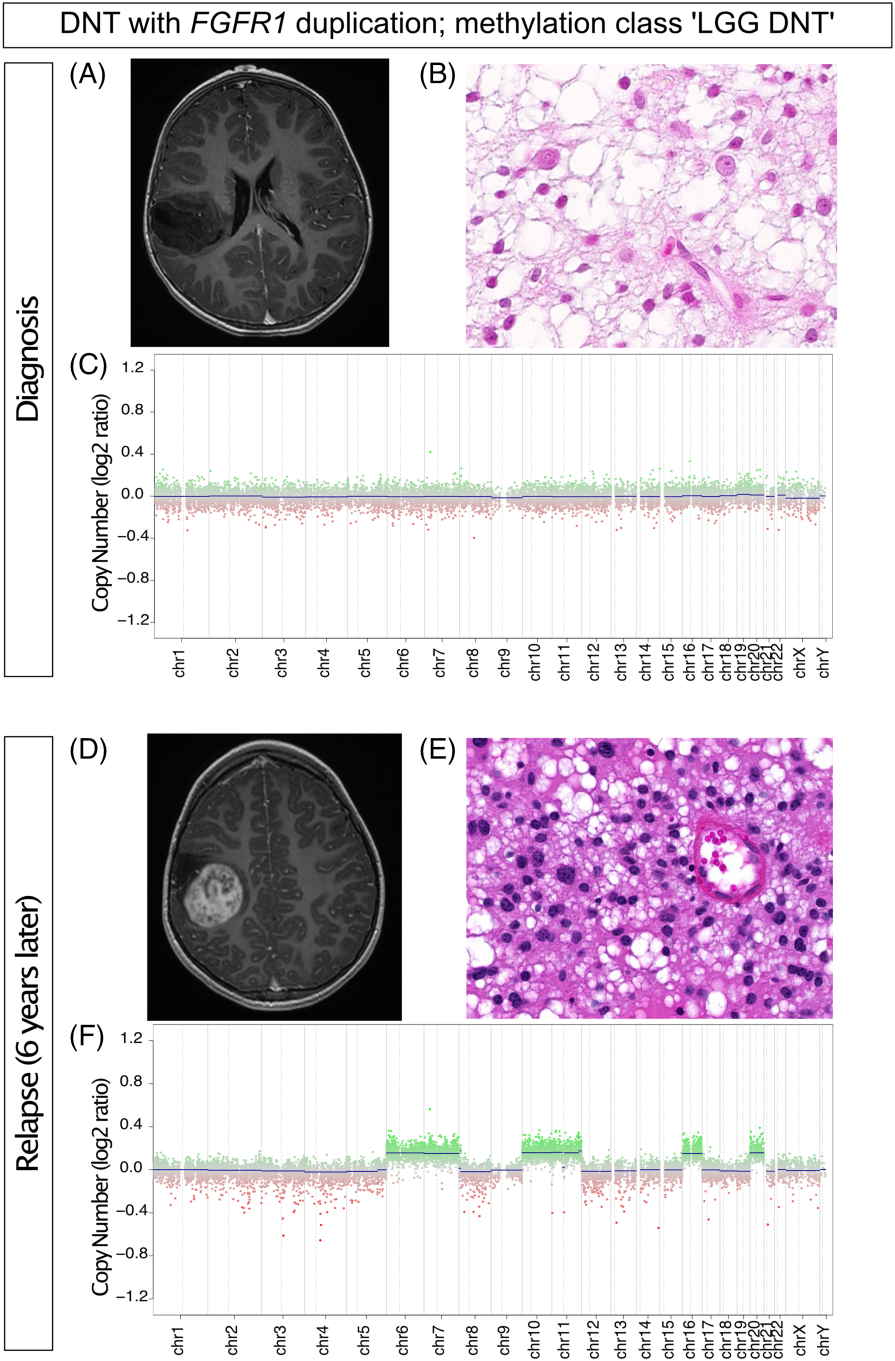
**Histopathological and copy number changes at progression in a DNT with FGFR1 duplication** *.* (top panel) Characteristics of the tumour at diagnosis: axial T1‐weighted pre‐operative MR image showing a hypointense signal without contrast enhancement (A), HES showing typical complex DNT (B), copy‐number profile showing no copy number alteration (C); (bottom panel) Characteristics of the tumour at relapse: axial T1‐weighted pre‐operative MR image showing mass arising in the operative cavity with contrast enhancement (D), HES showing cytonuclear atypia (E), copy‐number profile showing gain of chromosomes 6, 7, 10, 11, 16 and 20 (F). Magnification X200 (Figure 2B, E)

At second surgery, 8 of the 21 tumours with histology available at diagnosis displayed histopathological changes (38%), including increase cell density, emergence of cytonuclear atypia, and necrosis and vascular proliferation. Interestingly, these histopathological modifications aligned with changes in the copy number profile (Figure 2). WES data were available on paired tumours for two patients with progressive DNT (DNT_9 and DNT_47), and no additional variant was found at recurrence.

DNA methylation analysis at diagnosis was available for 16/25 of the progressive tumours; all were classified as “LGG, DNT” with the exception of two cases with a low tumour cell content (calibrated score for tissue control methylation class). Additionally, for the other nine cases, DNA methylation array data were available only at a subsequent operation: eight assigned to “LGG, DNT” methylation class and one tumour unassigned (low calibrated score). DNA methylation profiling was available for eight paired tumours with no significant change in the methylation class between the initial surgery and the surgery at progression (supporting information Tables S1 and S3).

**FIGURE 3:**
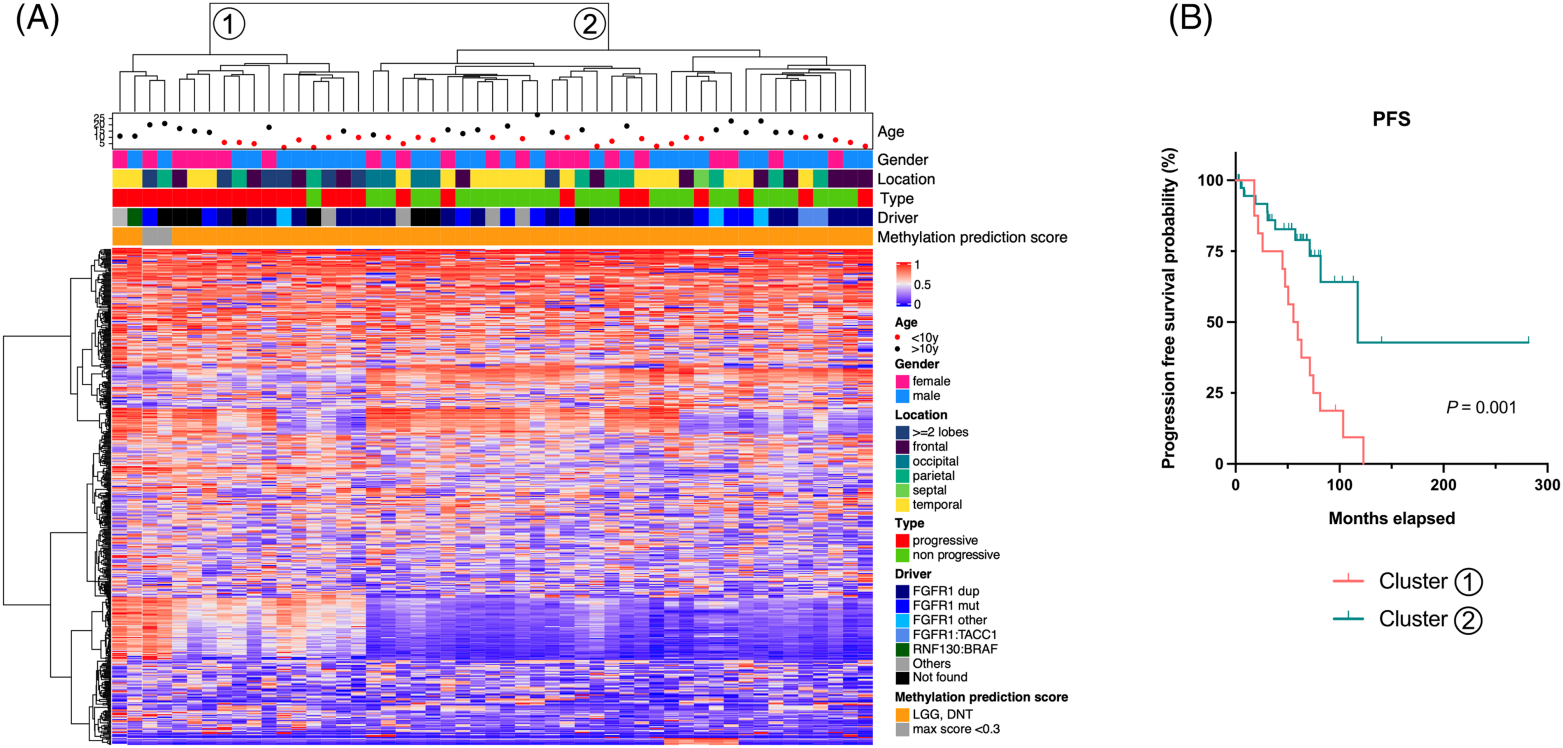
(A) **Unsupervised hierarchical clustering based on 51 DNT methylation profiles** *.* Unsupervised hierarchical clustering revealed two main clusters named 1 and 2. Histopathologic and genetic correlates are shown and colour coded. The heatmap (*red* increase methylation, *blue* decreased methylation) showed distinct CpG methylation level. Cluster 1 was broadly enriched in progressive DNTs. (B) **Survival analysis**. Kaplan–Meier estimates of the progression‐free survival (PFS) stratified by DNA methylation Cluster 1 or Cluster 2. DNTs from Cluster 1 are associated with a higher risk of progression than DNTs from Cluster 2

Unsupervised hierarchical clustering of DNA‐methylation data from all specific DNTs of the cohort (i.e., excluding those morphologically diagnosed as “non‐specific”) identified two clusters (Cluster 1 and Cluster 2). Interestingly, Cluster 1 was broadly enriched with samples from progressive DNTs (Figure 3A) suggesting that progressive specific DNTs have some differences in the DNA methylation profile. Comparing Cluster 1 and Cluster 2, a significant difference was observed for progression free survival (*p* = 0.001), suggesting that DNA methylation profiling might convey prognostic information (Figure 3B) but not for overall survival (supporting information Figure S3). No association between tumour location, age, sex, histological, immunophenotypical or molecular characteristics and the two clusters was identified (data not shown).

### Deciphering non‐specific DNT

**FIGURE 4:**
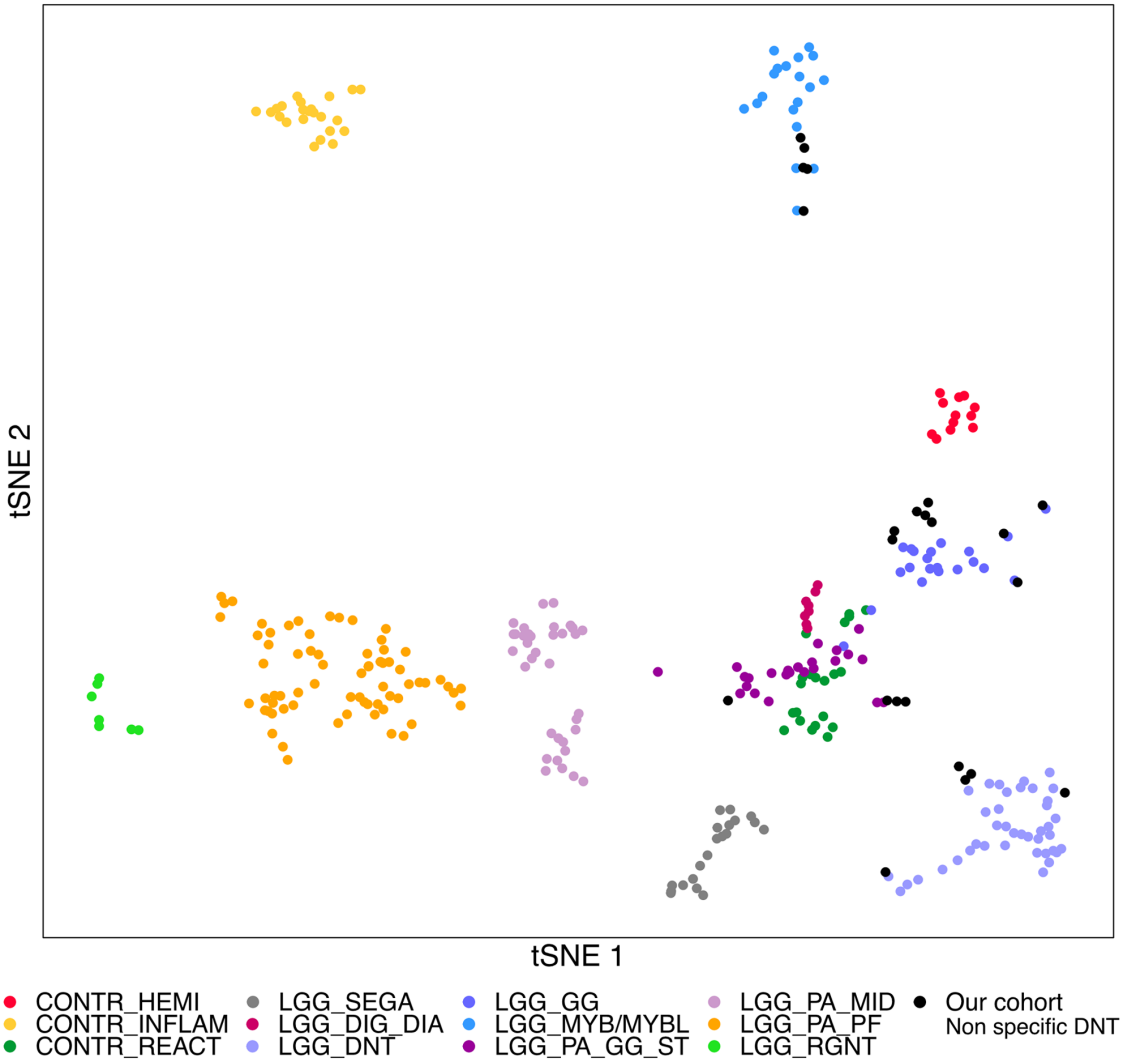
**Methylation‐based t‐SNE distribution of 23 “non‐specific” DNTs.** The 23 “non‐specific” DNTs with DNA methylation data available were compared with 244 reference low‐grade gliomas samples cohort belonging to 9 methylation classes and 74 control samples from the German Cancer Research Center (DKFZ) [32]. The 51 cases of this study are indicated as black dots

Twenty‐four tumours for which the histopathological review did not identify a specific glioneuronal element were classified as “non‐specific” DNT according to the current WHO classification criteria. DNA methylation profiling classified three cases as “LGG, DNT” with a high calibrated score, including two harbouring *FGFR1* variants. Despite a gross total resection of both tumours, extensive histopathological review did not identify with certainty a specific glioneuronal element. Two additional tumours obtained a calibrated score of 0.7 for this methylation class, including one harbouring a *RNF130:BRAF* fusion. A two‐dimensional t‐SNE projection alongside 244 LGGs and LGNTs from the Heidelberg reference cohort showed that all these five tumours clustered within the “LGG,DNT” group (Figure 4 and supporting information Table S2).

Five tumours were classified as “LGG, MYB/MYBL” by DNA methylation profiling. Copy‐number analysis revealed genomic events involving *MYBL1* in four tumours and *MYB* in one tumour. The presence of a *MYB/MYBL1* structural variant was confirmed in four cases (three *MYBL1*, one *MYB* and one failed). All four *MYBL1* tumours shared the same histological pattern as described in isomorphic glioma, with regular cells scattered in a fine bubbly neuropil [37] (supporting information Figure S4). Although structural variants involving *MYB* have been described to be associated with angiocentric glioma, an extensive histopathological review of the *MYB* tumour did not detect any angiocentricity.

BRAF V600E screening found four positive cases. Additional events involving *BRAF* were identified in four cases by RNA sequencing and FISH analysis using a break‐apart probe. RNA sequencing revealed a *RNF130‐BRAF* fusion in one case, whereas FISH showed a split of signal in the three other cases (no RNA sequencing performed). In two of these tumours, duplication of the 3′ red signal was observed, suggesting the presence of the common *KIAA1549:BRAF* tandem‐duplication characteristic of pilocytic astrocytoma. One tumour showed a loss of the 5′ signal suggesting a *BRAF* rearrangement. Copy‐number analysis from the DNA methylation assay suggested an event in the *BRAF* region in all four cases. DNA methylation profiling classified the *BRAF*‐mutated tumours as “LGG, GG”. Two *BRAF*‐rearranged tumours fell in the “LGG, PA/GG ST” class and no class was assigned with certainty to the two remaining tumours with a *BRAF* rearrangement. Both tumours harbouring 3′ *BRAF* duplication were cortical lesions in infants (1 and 2 years old). Review of the imaging revealed strong similarities between the tumours. They were both large temporal lesions with a homogeneous hypoT1 signal and a strong hyperT2 signal. Unlike pilocytic astrocytoma, neither tumour showed contrast enhancement or a cyst. Likewise, both tumours shared the same histopathological features: isomorphic astrocytic cells with a slightly microcystic appearance without nodule formation and without tumour cells infiltrating into the cortex. Typical histopathological features of pilocytic astrocytoma, such as Rosenthal fibres, were not observed (supporting information Figure S5).

All three BRAF V600E tumours lacked typical histopathological features of ganglioglioma, especially ganglion cells. Similarly, typical histopathological features of pilocytic astrocytoma or ganglioglioma were not observed in the *BRAF*‐rearranged tumours including the *RNF130‐BRAF* fused tumour. They are composed of mildly atypical glial cells with bland, spindle‐shaped nuclei.

RNA sequencing detected two fusions between *FGFR2* and *INA*. Using a break‐apart probe FISH, we detected two additional tumours with an *FGFR2* rearrangement, supported by the copy number profile analysis. Three of these tumours were classified as “LGG, GG”, and one was not assigned to any class with certainty. As described in PLNTY, histology was heterogeneous [38]. Two shared the same oligo‐like pattern, whereas one tumour presented a fibrillary architecture with astrocytic features. Calcification was observed in three cases. All four tumours were characterised by a remarkably strong extravascular CD34 positivity (supporting information Figure S6).

No recurrent alteration was detected in five tumours, but further analyses detected potential driver events. DNA methylation analysis was available for four of these cases. One was classified as “LGG, GG” and another one as “LGG, DNT”. DNA‐methylation profiling failed to classify with certainty two cases (supporting information Table S2 and S3).

**FIGURE 5:**
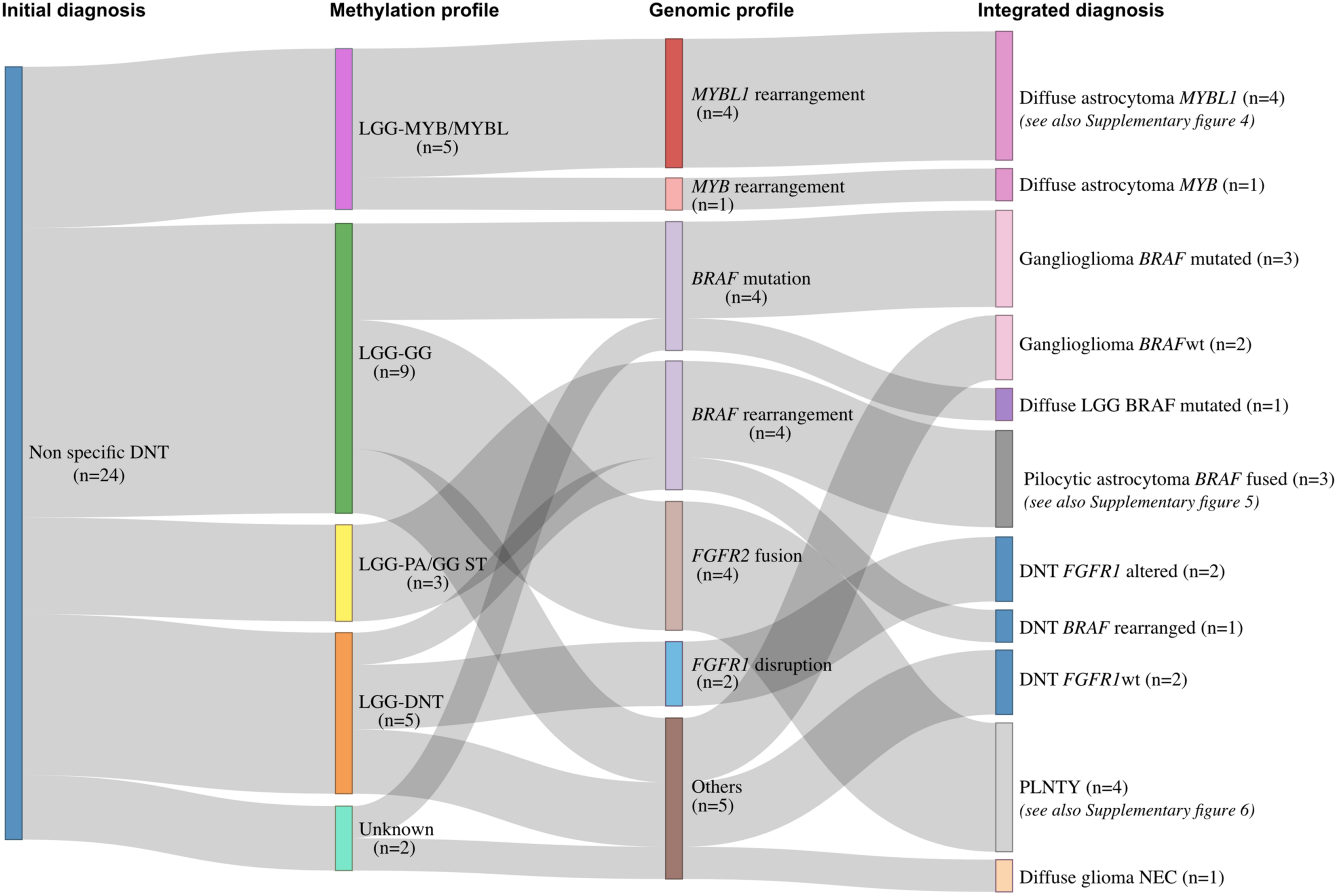
**Final data integration results in 24 “non‐specific” DNTs** *.* The integration of data from DNA methylation array to the histopathological diagnosis and molecular data helped establishing final diagnoses showing that the WHO tumour group “non‐specific” DNTs encompassed several morphomolecular entities. LGG = low‐grade glioma; GG = ganglioglioma; PLNTY = polymorphous low‐grade neuroepithelial tumour of the young; NEC = not elsewhere classified; wt = wild type

In summary, the integrative analysis enables one to refine this tumour group formerly named “non‐specific DNT” into more precise diagnoses of recently described entities (Figure 5).

## DISCUSSION

Most of the genomic alterations identified in paediatric glioneuronal tumours have been discovered in that last 5–10 years, resulting in recent and extensive restructuring of their classification, which has been updated in the sixth Consortium to Inform Molecular and Practical Approaches to CNS Tumour Taxonomy‐Not Official WHO (cIMPACT‐NOW) update and integrated into the 2021 WHO CNS classification [39, 40]. DNT is the second most common glioneuronal tumour and accounts for 5 to 20% of histopathological diagnoses in epilepsy surgery [3]. This range of reported incidence could be explained by a low inter‐observer diagnostic concordance, even among experienced neuropathologists [41], due to sampling artefacts, heterogeneity of the cyto‐architectural distribution of the glial and neuronal components, and also the recent description of various newly recognised histomolecular tumour types. However, before 2016, little molecular data were available for DNTs. Two studies published simultaneously in 2016, reported, for the first time, a high frequency of *FGFR1* disruption in DNTs, including mutation, ITD and fusion [36, 42, 43]. In our cohort of 58 specific DNTs, we found a similar frequency (68%). Whilst *FGFR1* alterations have been recently reported, BRAF V600E mutation has been described in DNT for a few years [9, 44, 45]. However, we did not detect any *BRAF* mutation in our cohort of specific DNTs as previously reported by Rivera et al. [42]. This could be explained by the fact that despite non‐specific DNT being described in the 2016 WHO classification, this histopathological subtype lacking the specific glioneuronal element remains controversial. It was used either rather as a generic term (all the clinical, radiological, morphological aspects of DNT except the glioneuronal element) or by numerous alternative terminologies used over time (DNT like, diffuse‐oligodendroglial tumour, glioneuronal tumour, NOS, paediatric oligodendroglioma and diffuse glioneuronal tumour). Consequently, due to terminology issues and the wide spectrum of histopathological features, there was significant heterogeneity in the published cohorts of DNTs and other epilepsy‐associated tumours, which some of which (but not all) will have included non‐specific DNTs, depending on preferences and convictions of each group.

No series with molecular data including non‐specific DNT has been published to date, in such a way that makes clear whether certain genomic alterations distinguish them from specific DNTs or from other LGNT. We studied 24 non‐specific DNTs. *BRAF* was the most commonly altered gene in the cohort (33%), followed by *MYBL1/MYB (20%)* and *FGFR2 (16%)*. We also found that a subset of “non‐specific” DNT had the classical DNT molecular hallmarks (*FGFR1* disruption and DNA methylation profile). Although these cases were fully resected, no specific glioneuronal element has been found even after a careful review. It could be suggested that sampling artefacts and/or the loss of the semi‐liquid mucoid specific element during the neurosurgical procedure explains this discrepancy. Our integrated analysis shows that non‐specific DNTs encompass a large spectrum of tumours, including recently described histomolecular types. Thus, “non‐specific DNT” could correspond to a generic term rather than to a distinct tumour entity, and greater efforts are necessary to harmonising the terminology of these tumours.

DNTs are WHO grade 1 tumours, classically depicted as stable, even in the event of partial surgical resection, and their prognosis depends primarily on the epilepsy‐related morbidity [11, 12, 13, 14]. Nevertheless, around 20 cases of progressive tumours or showing malignant transformation have been described in the literature out of more than 1000 DNTs published [15, 16, 17, 18, 19, 20, 21, 22, 23, 24, 25, 26]. However, no large series have investigated the progressive form of DNTs and no molecular data are available. Here, we analysed 25 tumours histologically and molecularly confirmed as DNT that showed radiological evidence of progression. DNA methylation classification confirmed the homogeneity of our cohort since the classifier calibrated scores observed were concordant with the histopathological diagnosis. To date, this cohort represents a unique cohort of progressive DNTs with molecular data. We did not identify specific recurrent genomic alterations in this group and found the same frequency of *FGFR1* disruptions than in non‐progressive DNTs. Increased numbers of CNA were detected at the second surgery in comparison with the original tumour, in parallel to the histopathological changes. This observation was previously reported in a case report describing a DNT showing malignant transformation 5 years after the first surgery [23]. These data support the idea that although DNTs are grade 1 tumours, they can go on to progress, as can be observed in other grade 1 paediatric brain tumours with MAP kinase alterations (ganglioglioma and pilocytic astrocytoma). The median follow‐up of the cohort was 7 years, with a median of 5 years between the first and the second operation. This confirms that local recurrence can occur several years after the first surgery (up to 9 years in our study). These data point out the relevance of long‐term clinical and imaging monitoring of these patients. Interestingly, our data tend to indicate that DNA methylation profiling could convey prognostic information and may represent a relevant biomarker in predicting a risk of recurrence. Presently, there are few prognostic factors in glioneuronal tumours. Of note, as in our study, two prognostic subtypes of diffuse leptomeningeal glioneuronal tumours (DLGNT) have been recognised based only on DNA methylation profiling (DLGNT‐ MC‐1 and MC2). DLGNT–MC‐2 is enriched for 1q gain and is associated with a shorter survival [46]. In DNTs, the cluster 1 is enriched with progressive form of DNT, but we did not find any clinical, radiological, histological or molecular characteristics associated with this epigenetic signature, and further studies are needed to validate these new data.

## CONFLICTS OF INTEREST

The authors declare no conflict of interest.

## ETHICS STATEMENT

All patients consented to participation in the Necker hospital tumour bank, and the study received ethical committee approval (ID‐RCB 2017‐A01535–48).

## AUTHOR CONTRIBUTIONS

MP and PV conceptualised the study. MP and PV performed pathological review. RS performed targeting sequencing. MD and DC performed RNA sequencing and WES. DFB and FF performed ddPCR analyses. MP collected the data. MP and PV completed the research and analysed the data. PV and RB supervised the study. MP designed the figures. MP wrote the original draft of the manuscript, and all authors revised it critically and approved the final version.

## Supporting information


Table S1

Table S2

Table S3

Table S4

Figure S1

Figure S2

Figure S3

Figure S4

Figure S5

Figure S6

## Data Availability

The data that support the findings of this study are available on request from the corresponding author. The data are not publicly available due to privacy or ethical restrictions.
