# Supplementary figures and images for: The genomic landscape of dysembryoplastic neuroepithelial tumours and a comprehensive analysis of recurrent cases
Source: Neuropathol Appl Neurobiol. 2022 Aug 9;48(6):e12834. doi: 10.1111/nan.12834 (PMC9542977; doi:10.1111/nan.12834)

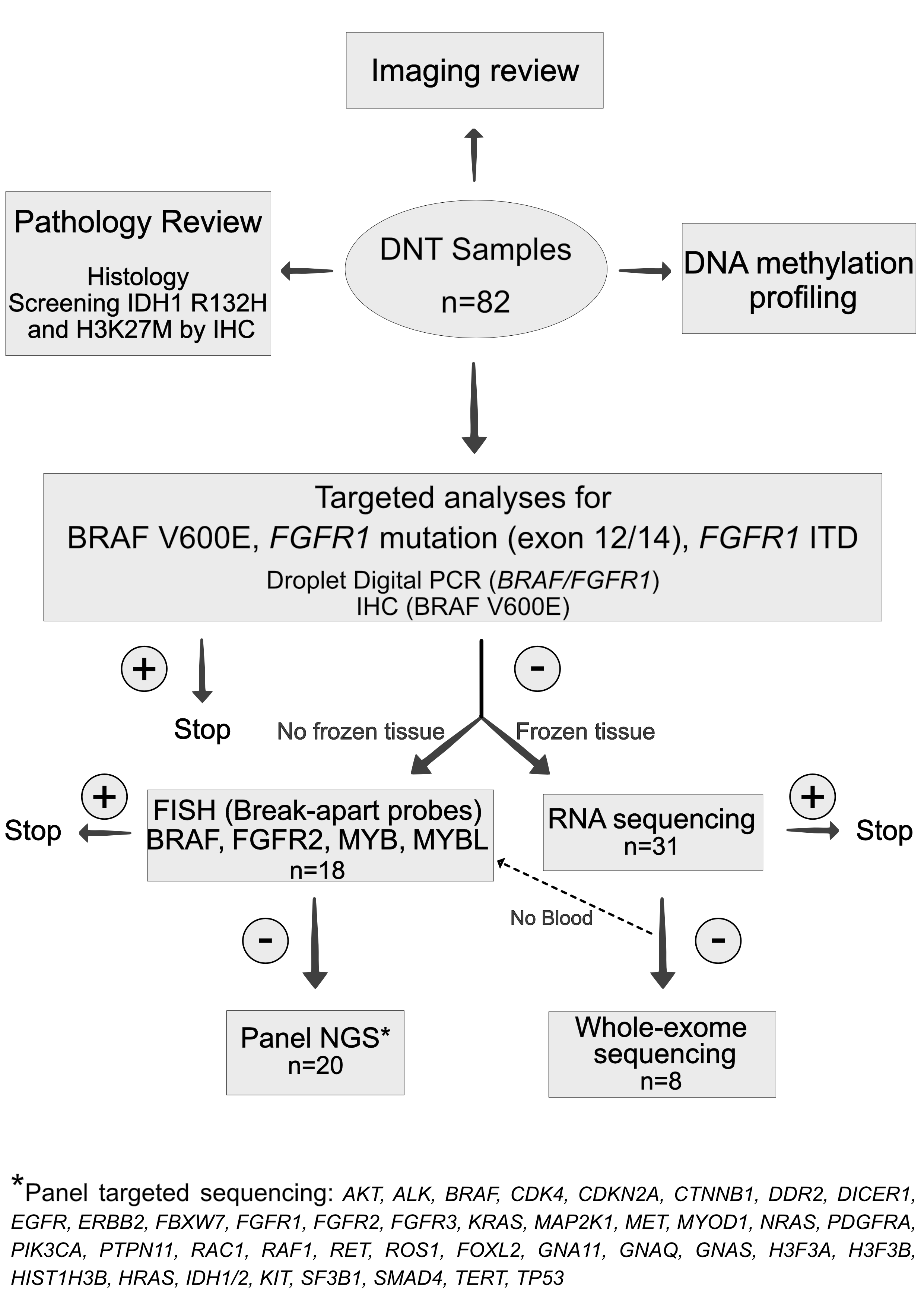

Supplement: Supplementary file 5 — Figure S1 [file NAN-48-0-s008.tiff]

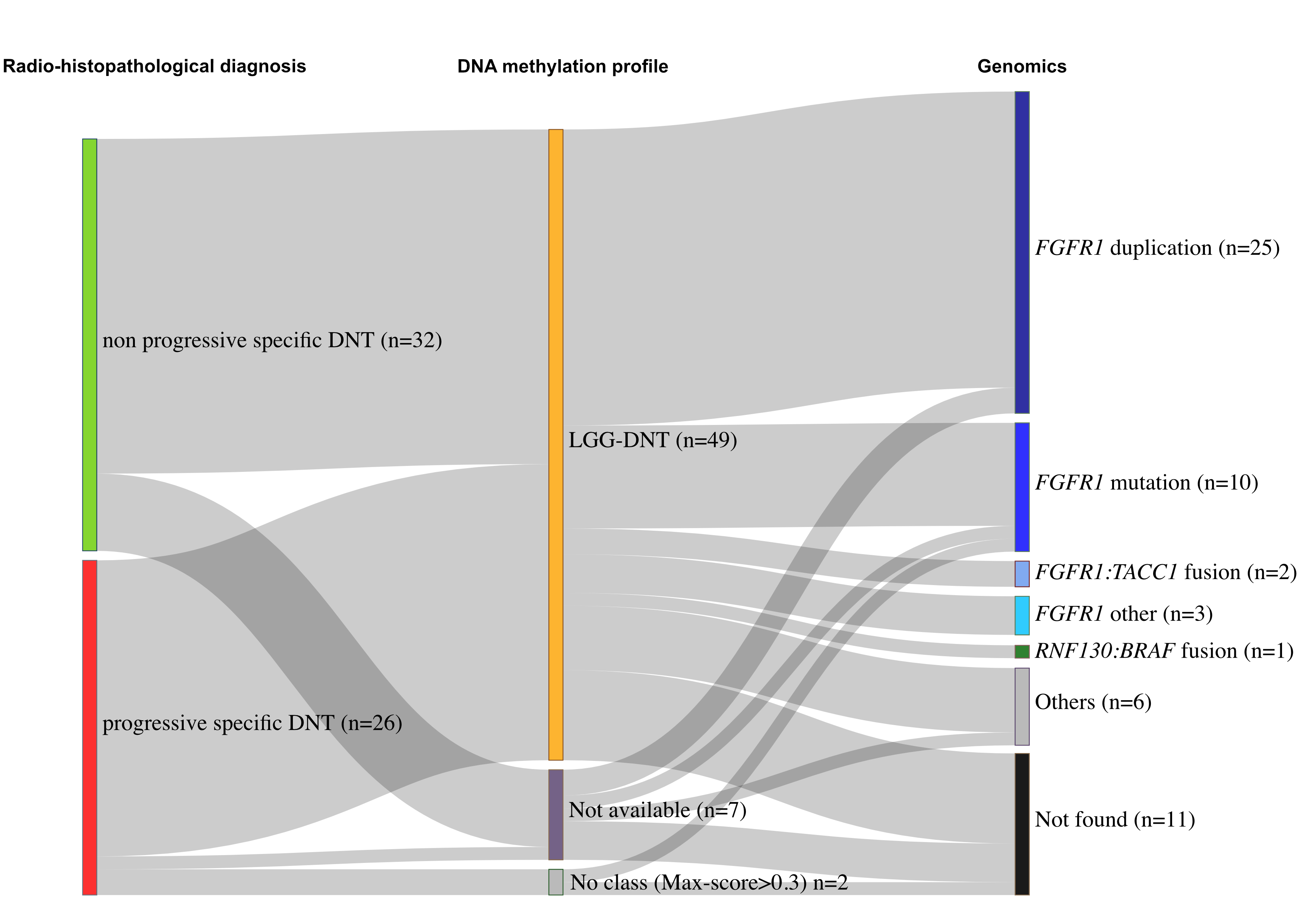

Supplement: Supplementary file 6 — Figure S2 [file NAN-48-0-s010.tiff]

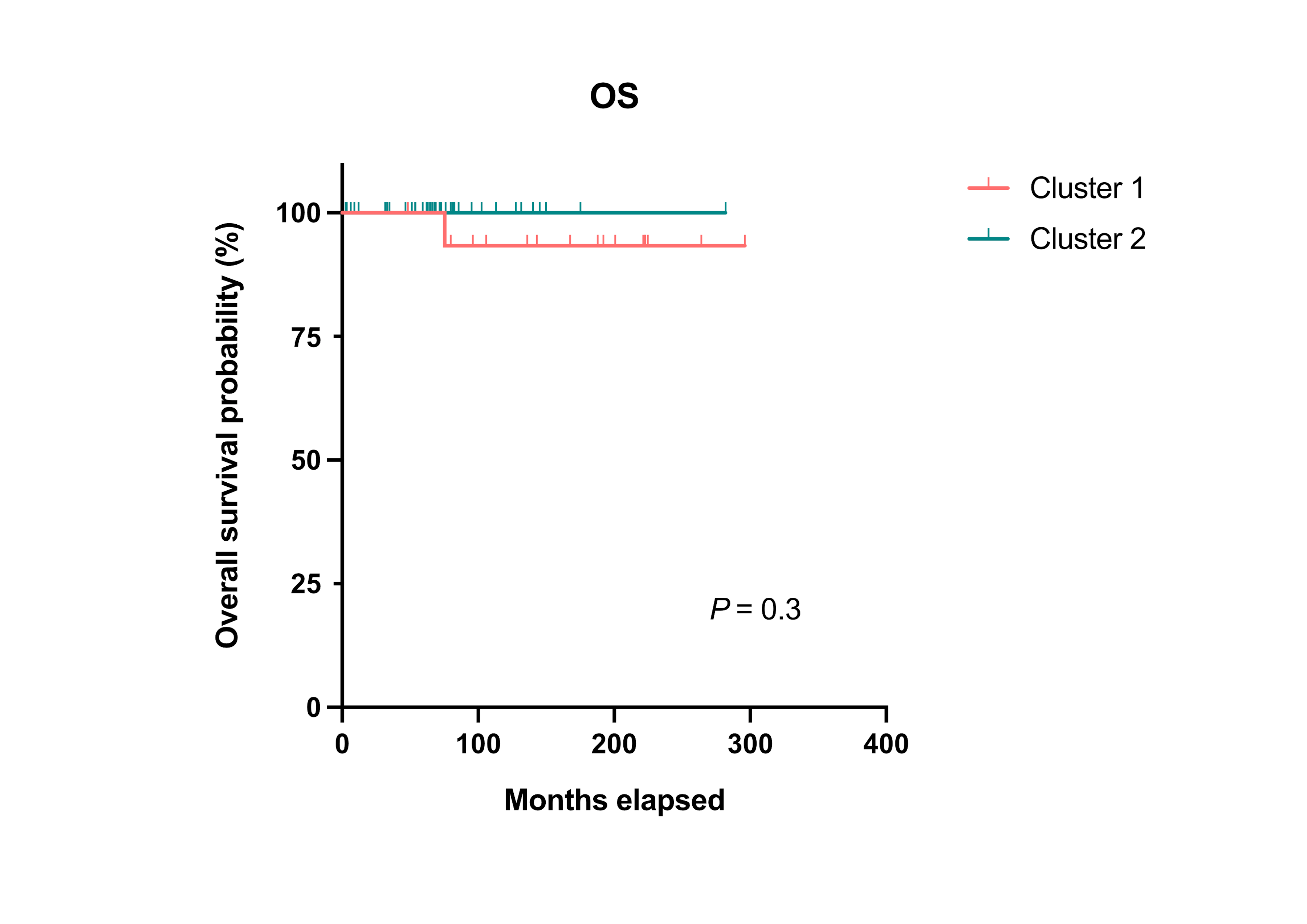

Supplement: Supplementary file 7 — Figure S3 [file NAN-48-0-s004.tiff]

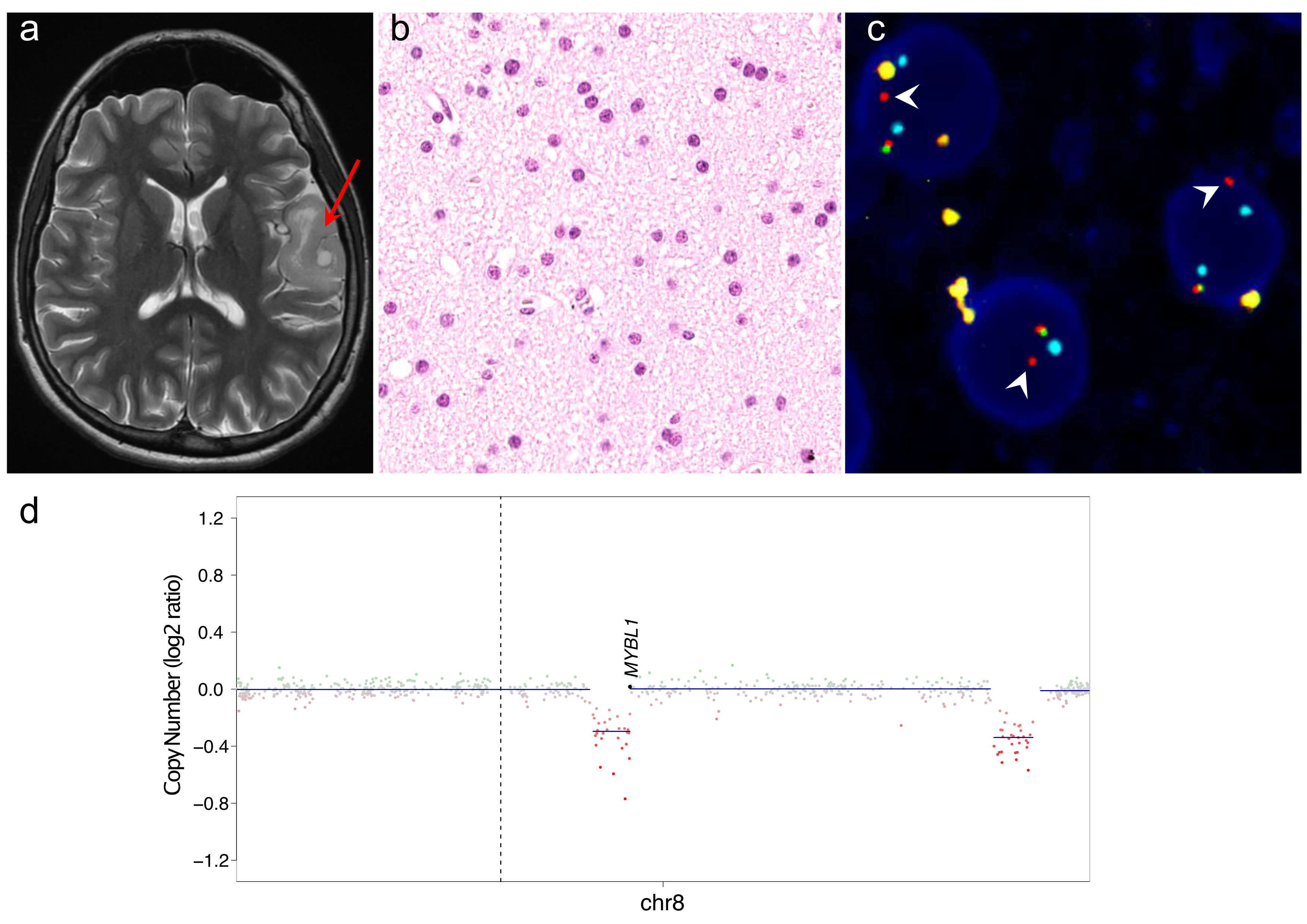

Supplement: Supplementary file 8 — Figure S4 [file NAN-48-0-s001.tiff]

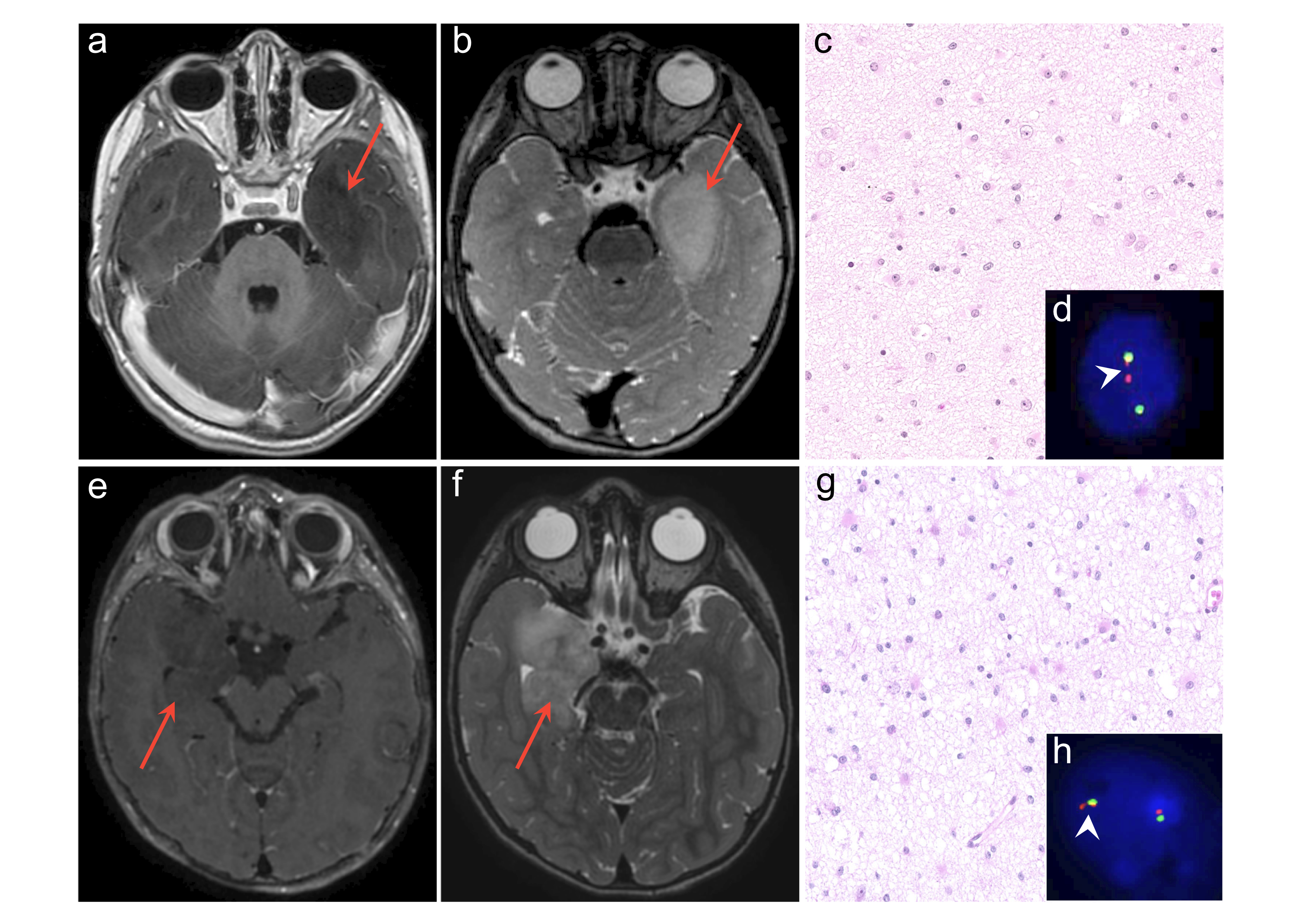

Supplement: Supplementary file 9 — Figure S5 [file NAN-48-0-s005.tiff]

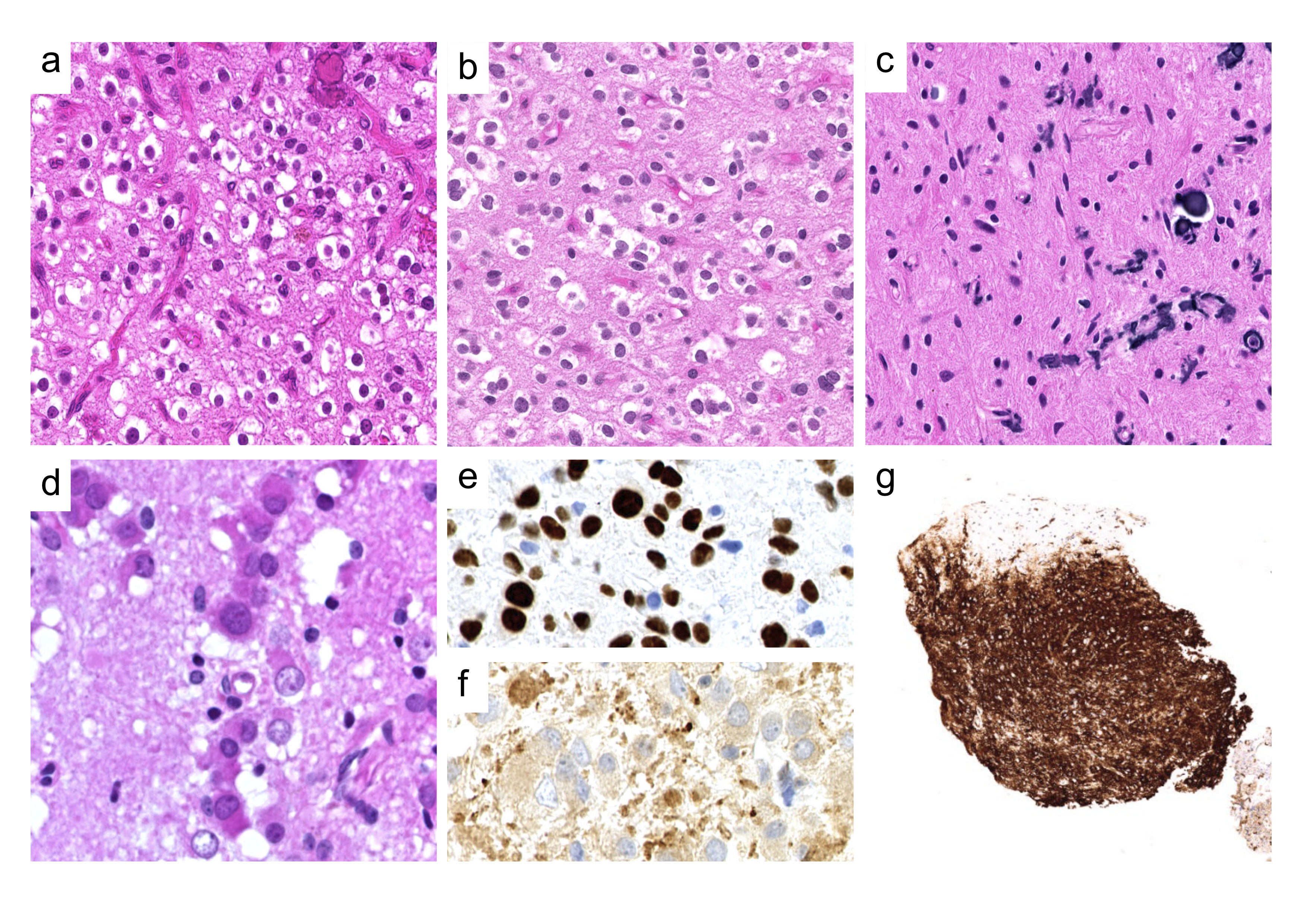

Supplement: Supplementary file 10 — Figure S6 [file NAN-48-0-s006.tiff]
